# Involvement of Autophagy in Cardiac Remodeling in Transgenic Mice with Cardiac Specific Over-Expression of Human Programmed Cell Death 5

**DOI:** 10.1371/journal.pone.0030097

**Published:** 2012-01-11

**Authors:** Lin An, Xiwen Zhao, Jian Wu, Jianguo Jia, Yunzeng Zou, Xizhi Guo, Lin He, Hongxin Zhu

**Affiliations:** 1 Bio-X Institutes, Key Laboratory for the Genetics of Developmental and Neuropsychiatric Disorders, Ministry of Education, Shanghai Jiao Tong University, Shanghai, China; 2 Institutes of Biomedical Sciences, Fudan University, Shanghai, China; I2MC INSERM UMR U1048, France

## Abstract

Programmed cell death 5 (PDCD5) is a cytosolic protein suppressing growth of multiple types of cancer cells through activating p53. We hypothesized that PDCD5 plays an essential role in cardiac remodeling and function. PDCD5 was significantly up-regulated in the hearts from mice subjected to angiotensin II treatment or transverse aortic constriction. Thus, we generated transgenic mice over-expressing human PDCD5 under the control of alpha myosin heavy chain promoter to examine the role of PDCD5 in cardiac remodeling. Transgenic founder died spontaneously displayed enlarged heart. The high PDCD5 over-expressing line (10-fold) showed reduced survival rate, increase in heart weight normalized to body weight. Real-Time RT-PCR analysis revealed fetal gene program was up-regulated. Echocardiography and histopathological examination showed characteristics of dilated cardiomyopathy and heart failure in transgenic mice. Western blot and immunohistochemistry analysis showed autophagy was dramatically increased in transgenic mice as compared to WT littermates control mice, while apoptosis remained unchanged. The enhanced autophagy in high over-expressing line was associated with significant increase in p53 activity and its downstream target damage-regulated autophagy modulator expression. The low over-expressing line (3.5-fold) appeared normal, but was more susceptible to angiotensin II-induced cardiac hypertrophy. This study is the first providing evidence that PDCD5 plays an important role in cardiac remodeling.

## Introduction

Programmed cell death 5 (PDCD5) was initially cloned from apoptotic TF-1 cells and currently known as a tumor suppressor candidate [1]–[4]. PDCD5 is up-regulated in various cells undergoing apoptosis and translocated from cytosol to nucleus to execute its apoptotic function [5]. Apoptotic potential of PDCD5 is linked with CK2 phosphorylation [6]. A recent study demonstrates that PDCD5 positively regulates Tip60, a transcriptional coregulator, which in turn, promotes p53 acetylation, leading to enhanced p53-dependent apoptosis [7]. It has also been shown that PDCD5 can enhance TAJ/TROY–induced paraptosis–like cell death [8]. In addition, PDCD5 plays an important role in the pathogenesis of rheumatoid arthritis [9]–[10]. However, nothing is known about the role of PDCD5 in cardiac remodeling and function.

Macroautophagy (refer to here as autophagy) is a highly conserved process of bulk protein degradation by lysosomes. Under basal or certain stress conditions such as nutrient starvation, autophagy promotes cell survival by eliminating misfolded proteins and organelles and provides energy and amino acids for the cells [11]–[12]. However, excessive levels of autophagy lead to autophagic cell death [13]–[15]. A growing body of evidence has shown that enhanced autophagy is associated with numerous diseases such as cancer, neurodegenerative disorders, myopathies and infectious deseases [16]–[17]. Recent studies demonstrate that dysregulation of autophagy contributes to the pathogenesis of many forms of heart diseases and its function is dose and context dependent [18]–[22]. Autophagy can be induced by a number of stimuli and regulated by multiple signaling pathways including p53 [11]–[12], [23]–[25]. Regulation of autophagy by p53 is dependent on its subcellular localization [23]. On the one hand, nucleus p53 stimulates autophagy through transcriptional effects including transactivation of damage-regulated autophagy modulator (DRAM) [24]. On the other hand, cytoplasmic pool of p53 represses autophagy through poorly characterized mechanisms [25]. Given the regulatory associations between PDCD5, p53 and autophagy, we hypothesize that PDCD5 is essential for cardiac remodeling and function through regulating autophagy.

In this study, we created transgenic mice with cardiac specific over-expression of human PDCD5 to investigate the role of PDCD5 in cardiac remodeling and function. We found that myocardial high PDCD5 over-expression results in dilated cardiomyopathy and heart failure accompanied by dramatically enhanced autophagy, which is associated with increased p53 activity. Transgenic mice with low over-expression of PDCD5 are more susceptible to angiotensin II (Ang II)-induced cardiac hypertrophy. Our study provides the first evidence that PDCD5 contributes to the cardiac remodeling and function through upregulating autophagic activity.

## Results

### PDCD5 is up-regulated in cardiac hypertrophy

**Figure 1:**
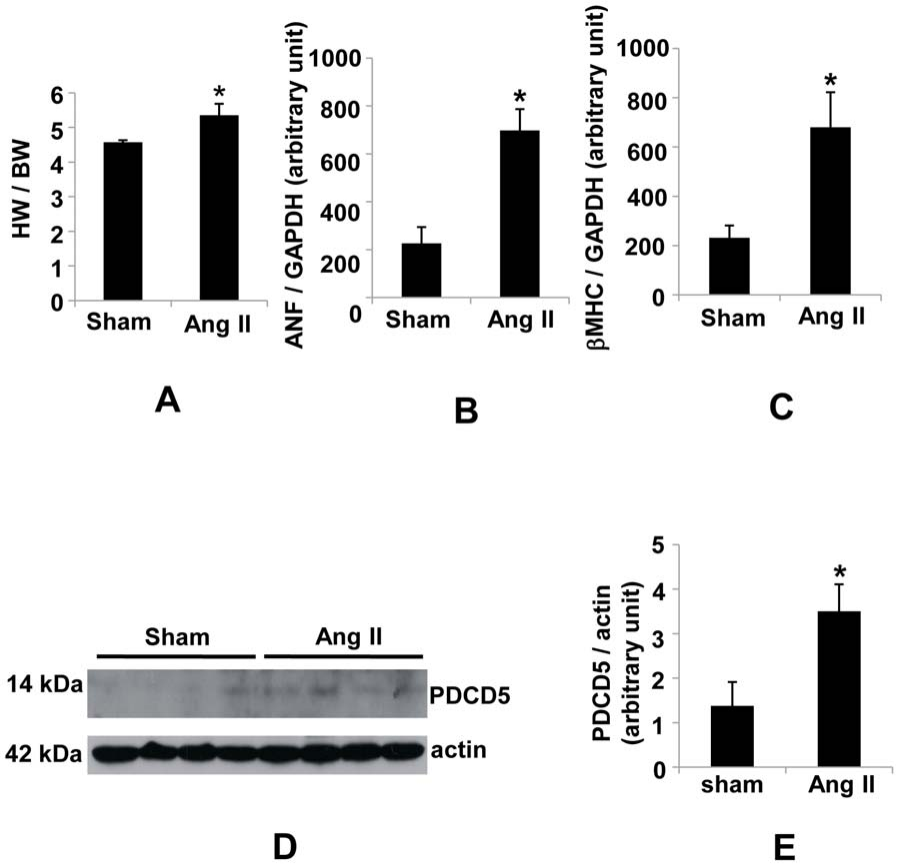
Up-regulation of PDCD5 in Ang II-induced cardiac hypertrophy. (A), HW∶BW ratio showing significant increase in cardiac mass in mice treated with Ang II for 2 weeks compared to sham control mice. (B) Expression of ANF was determined by quantitative Real-Time RT-PCR analysis in cDNA samples derived from hearts of mice treated with Ang II and sham control. (C), Expression of βMHC was determined by quantitative Real-Time RT-PCR analysis in cDNA samples derived from hearts of mice treated with Ang II and sham control. (D), Representative western blot of PDCD5 and internal control actin proteins in heart extracts from 8–week-old male mice with sham or Ang II treatment. (E), Quantitative analysis revealed that PDCD5 levels were up-regulated in hearts from mice treated with Ang II (n = 4) as compared to sham control mice (n = 4). *P<0.05, sham vs. Ang II treatment.

Previous study showed that PDCD5 mRNA was expressed at considerable levels in adult heart [1]. To determine whether PDCD5 may be involved in cardiac remodeling, we first examined PDCD5 protein levels in heart extracts from adult mice subjected to 2-week Ang II treatment. In this hypertrophic model, the heart-to-body weight ratio was significantly increased in Ang II-treated mice (5.3±0.1, n = 15) compared to sham control (4.7±0.1, n = 12; *p*<0.01) (Fig. 1A). Atrial natriuretic factor (ANF) and beta myosin heavy chain (βMHC), two molecular markers for cardiac hypertrophy, were significantly increased in Ang II-treated mice (Fig. 1B–C). Assessment of PDCD5 protein expression levels showed significant elevation in Ang II-induced hypertrophied hearts (Fig. 1D–E). Similarly, PDCD5 protein was significantly increased in heart from mice subjected to acute pressure overload for 2 weeks by transverse aortic constriction (Figure S1A–B). These data suggest PDCD5 may be involved in cardiac remodeling.

### Generation of alpha-MHC-hPDCD5 transgenic mouse line

**Figure 2:**
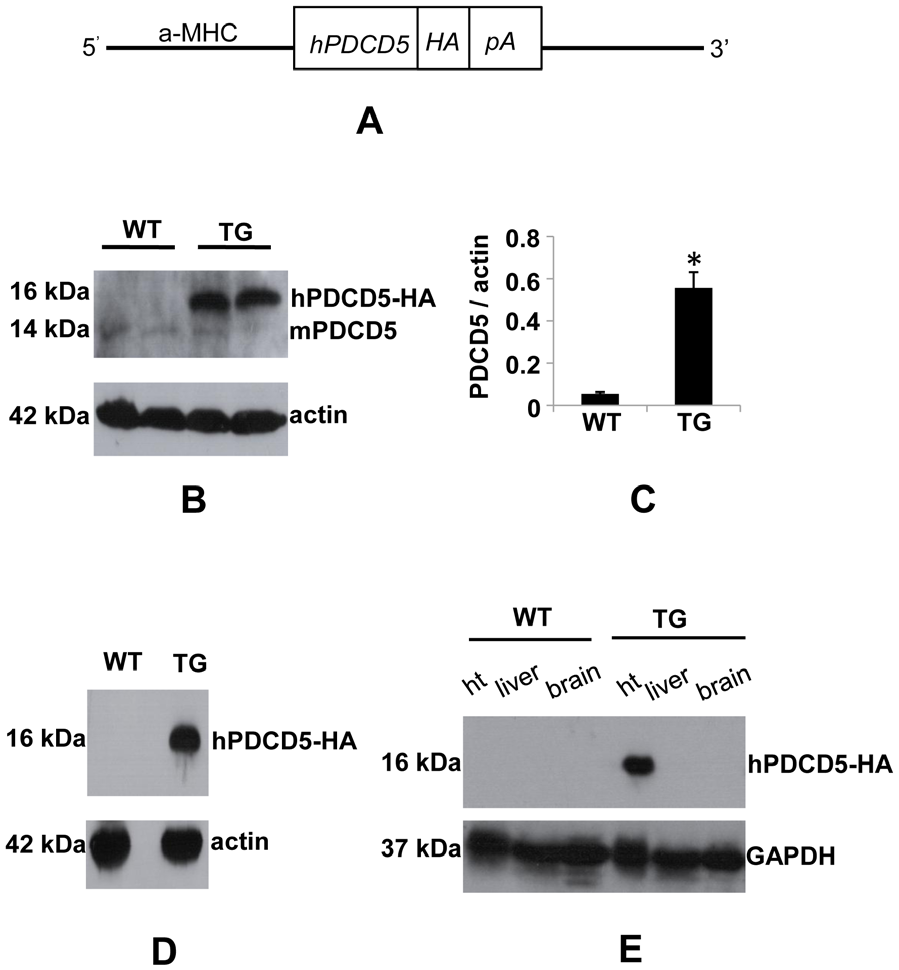
Generation of transgenic mice with cardiac specific over-expression of hPDCD5. (A), Schematic representation of the transgene construct of hPDCD5 cDNA under the control of the cardiac-specific alpha-MHC promoter (not at scale). (B), Representative western blot of hPDCD5 protein with PDCD5 antibody in heart extracts from high over-expressing line and WT control mice. Asterisk indicates endogenous PDCD5. (C), Densitometric analysis of PDCD5 immunoblots. (D), Representative western blot of hPDCD5 protein with HA antibody in heart extracts obtained from high over-expressing line and WT control mice. (E), Representative western blot of hPDCD5 protein with HA antibody in heart, liver, and brain extracts obtained from high over-expressing line and WT control mice.

**Figure 3:**
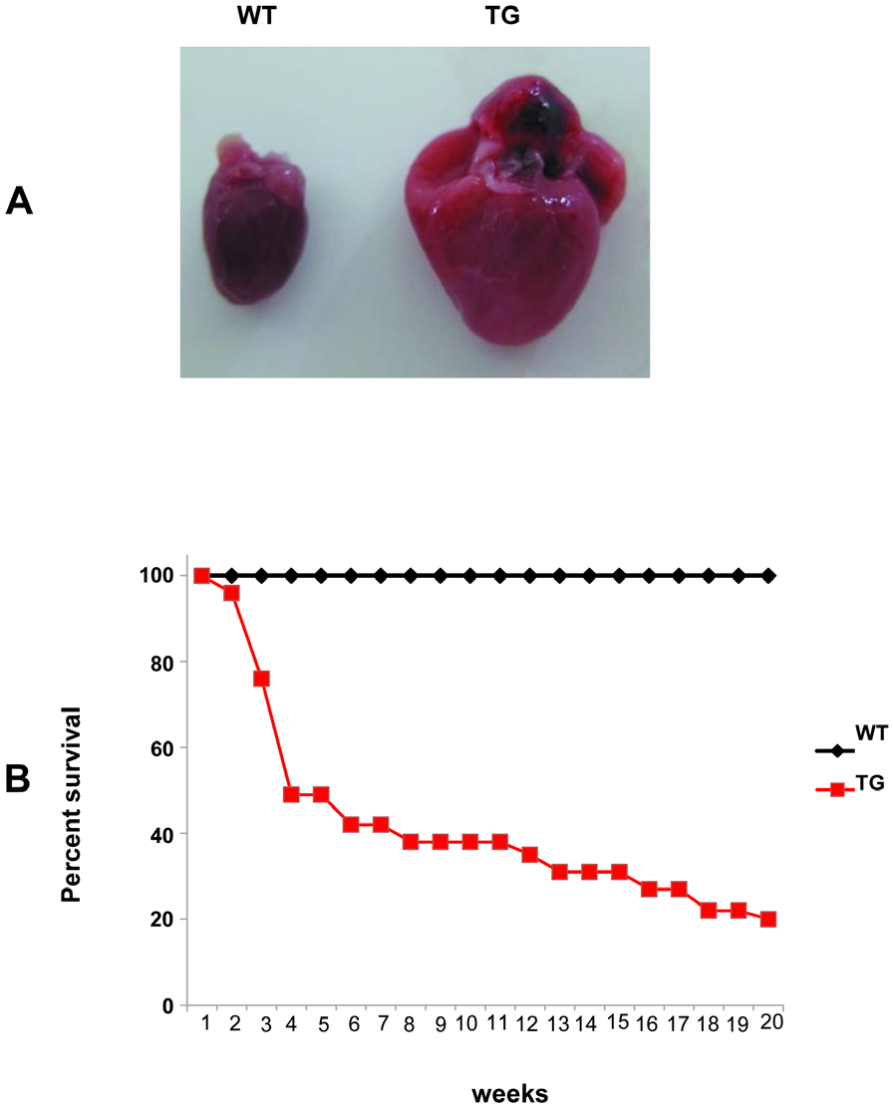
The effect of hPDCD5 over-expression on the survival of mice. (A), Autopsy of transgenic founder 41 showing dramatically enlarged heart. (B), Kaplan-Meier Survival Curves of high over-expressing line (red line) and WT littermate controls (black line). (n = 50 for WT, n = 65 for TG).

To define the potential role of PDCD5 in cardiac remodeling, we generated transgenic mice over-expressing human PDCD5 under the control of alpha myosin heavy chain promoter (Fig. 2A), which directs expression specifically to postnatal ventricular cardiomyocytes [26]–[28]. Five founders identified by PCR genotyping were maintained to generate transgenic lines. Two founders (number 19 and number 41) died spontaneously before successful mating. Autopsy of founder 41 showed enlarged heart (Fig. 3A). One founder (number 39) was not able to transmit transgene to the next generation. Founder 32 and founder 50 successfully produced progeny positive for the presence of transgene. Western blot with PDCD5 polyclonal antibody revealed hPDCD5 protein abundance was 10-fold higher (high over-expressing line) compared to endogenous PDCD5 protein in transgenic line 32 (Fig. 2B–C). Accordingly, Real-Time RT-PCR showed significant increase in PDCD5 mRNA in high over-expressing line (Figure S2). Furthermore, western blot with anti-HA antibody detected hPDCD5-HA fusion protein in heart (Fig. 2D), but not in other tissues such as liver and brain (Fig. 2E). In transgenic line 50, PDCD5 protein abundance was increased by 3.5 folds (low over-expressing line) in hearts as revealed by western blot analysis (Figure S3A). The heart-to-body weight ratio and cardiac function were comparable to WT littermate control mice (Figure S3B–C, Table S1).

### Cardiac specific high over-expression of hPDCD5 in transgenic mice results in reduced survival

High PDCD5 over-expressing line was born at Mendelian ratio. However, the transgenic mice started to die at the age of 3 weeks, and few were able to survive to six months of age (Fig. 3B). No difference in survival was shown between male and female mice. Mice dissection performed on anesthetized moribund mice showed obvious pulmonary edema, elevated lung weight normalized to body weight and enlarged hearts as compared to WT littermate control mice (Figure S4A–B), indicating transgenic mice developed heart failure.

### High PDCD5 over-expressing line develops dilated cardiomyopathy and heart failure

**Figure 4:**
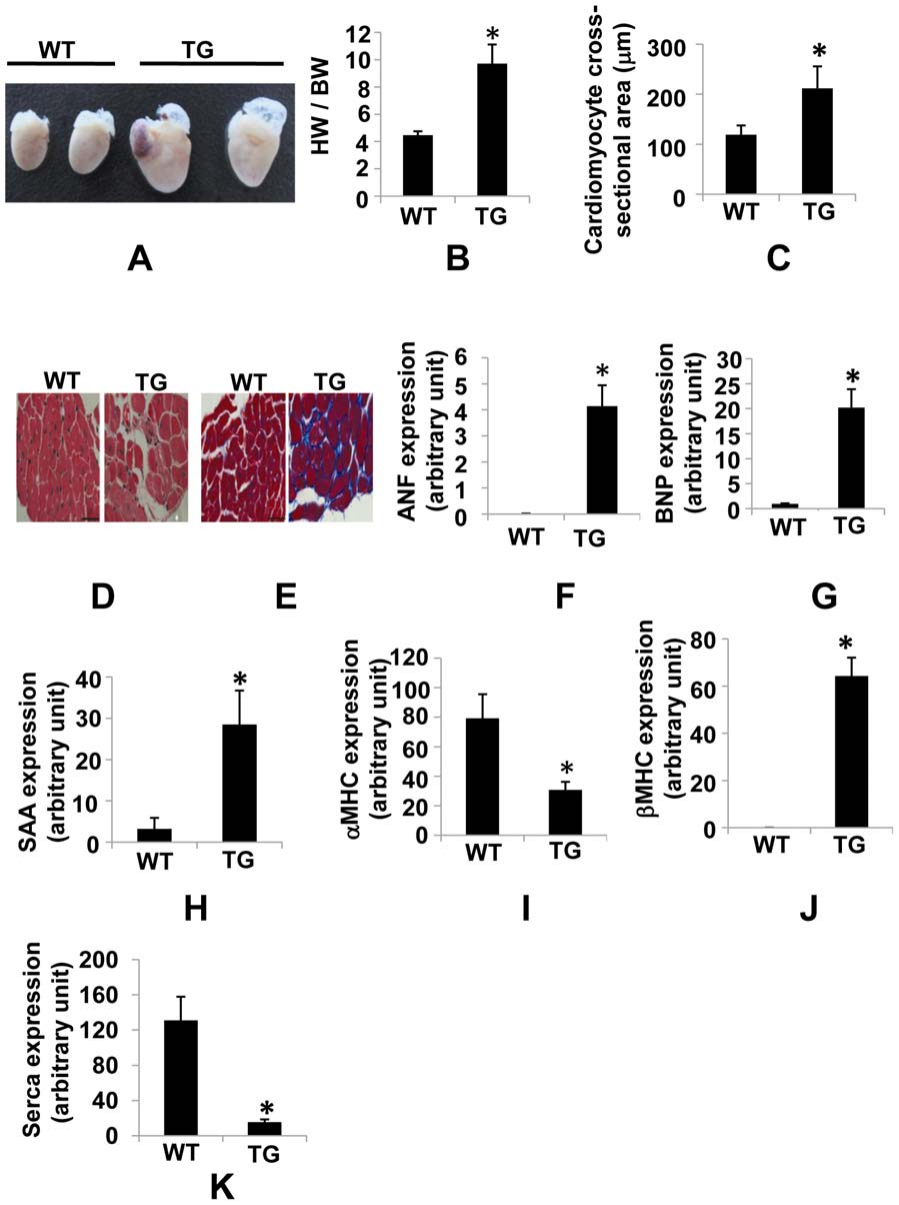
Cardiac remodeling in high PDCD5 over-expressing line. (A), Cardiac specific high over-expression of hPDCD5 results in enlarged hearts (3 months old). (B), HW∶BW ratio showing significant increase in cardiac mass in high over-expressing line compared to the WT control mice (n = 6 for TG, n = 6 for WT). (C), Measurement of two-dimensional cardiomyocyte cross-sectional area showing significantly enlarged cells in high over-expressing line compared with WT control mice (n = 4 for TG, n = 4 for WT). *P<0.05, WT vs. TG. (D), Histological analysis with H&E staining on heart sections. Bar, 50 µm. (E), Masson's trichrome staining showing collagen depositon in left ventricle of high over-expressing line. Bar, 50 µm. Expression of ANF (F), BNP (G), SAA (H), αMHC (I), βMHC (J) and SERCA (K) was determined by quantitative Real-Time RT-PCR analysis in cDNA samples derived from heart of high over-expressing line and WT control mice (n = 4 for TG, n = 4 for WT). Expression levels were normalized to GAPDH. Experiments were performed twice in triplicate with similar results. *P<0.05, WT vs. TG.

**Figure 5:**
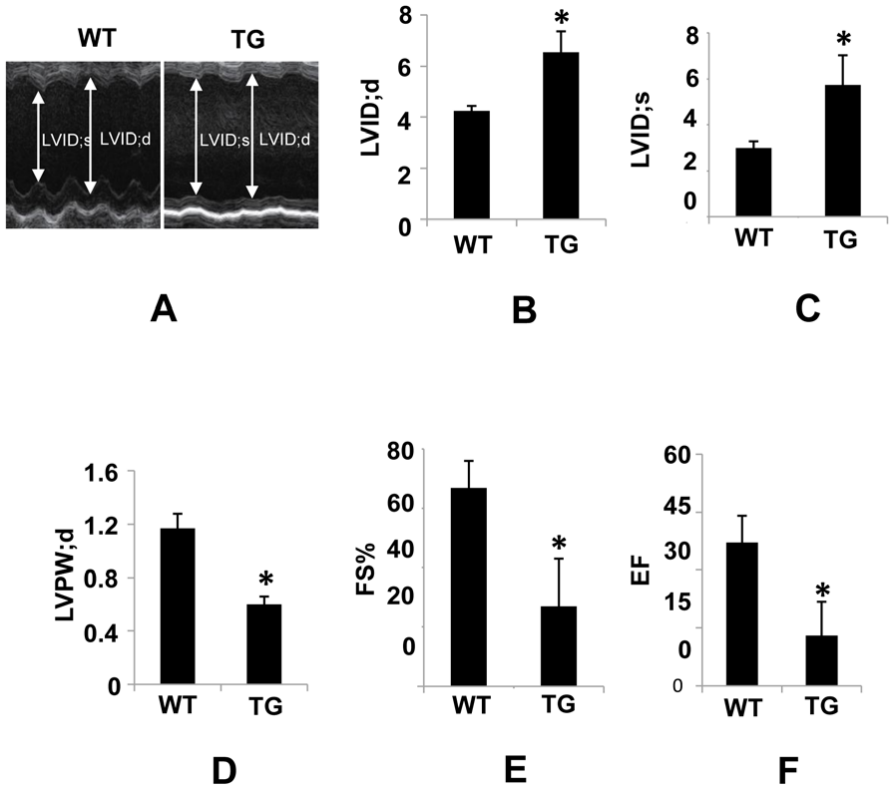
Echocardiographic analysis of cardiac function of high over-expressing line. (A), Representative M-mode echocardiography images in 3-month-old mice. (B)–(C), LV end-diastolic diameter (LVID;d) and LV end-systolic diameter (LVID;s) were significantly increased in high over-expressing line compared to WT control mice. (D), Left ventricular posterior wall (LVPW) was significantly decreased in high over-expressing line compared to WT control mice. (E)–(F), % fractional shortening and ejection fraction were significantly diminished in high over-expressing line compared to WT control mice. *P<0.05, WT vs. TG (n = 5 for TG, n = 5 for WT, 3 months old).

We next investigated cardiac pathology in high over-expressing line. The heart-to-body weight ratio was significantly increased in mice (Fig. 4A–B), with no significant difference in body weight between transgenic mice and age-matched WT control (data not shown). Accordingly, morphological analysis showed individual cardiomyocyte was significantly larger in transgenic mice compared to WT control mice (Fig. 4C), suggestive of cardiac remodeling. Hematoxylin and Eosin (HE) staining showed reduced thickness of ventricular walls, dilated heart chambers and evidence of disarray of cardiomyocytes in ventricles of transgenic hearts (Fig. 4D, Figure S4B). Masson trichrome staining showed collagen deposition, a hallmark of cardiac hypertrophy and heart failure, was significantly increased in transgenic mice (Fig. 4E). Consistent with this finding, Real-Time RT-PCR revealed significant increase in TGF-B, type I collagen, type III collagen mRNA expression (data not shown). Although the fetal gene program such as ANF, brain natriuretic peptide (BNP), skeletal α-actin (SAA), a molecular marker of cardiac hypertrophy and heart failure, barely detectable in hearts from WT control mice, was expressed at considerable levels in transgenic hearts (Fig. 4F–H). Moreover, increase in βMHC mRNA expression was associated with decrease in alpha myosin heavy chain (αMHC) mRNA expression (Fig. 4I–J), indicating a shift in myosin heavy chain from alpha to beta isoform in transgenic hearts. In addition, the sarcoplasmic/endoplasmic reticulum calcium ATPase (SERCA), an established molecular marker of heart failure, was significantly reduced in transgenic hearts (Fig. 4K). To further determine the alteration of cardiac structure and cardiac function of transgenic mice, we performed echocardiography on mice at 3 months of age. In transgenic mice, LV end-diastolic diameter (LVID;d) and LV end-systolic diameter (LVID;s) were greater as compared to WT control mice (Fig. 5A–C). Left ventricular posterior wall thickness (diastole) (LVPW;d) was significantly reduced (Fig. 5D), suggesting the hearts were dilated in transgenic mice. LV function was severely impaired in transgenic mice as attested by a 65% decline in percent fractional shortening (FS%) (Fig. 5E) and a 75% reduction in ejection fraction (EF) (Fig. 5F), demonstrating transgenic mice developed heart failure. Consistent with these findings, Lung weight normalized to body weight was significantly increased in transgenic mice compared to WT control mice, indicative of lung congestion (data not shown). Taken together, high PDCD5 over-expressing line developed dilated cardiomyopathy and heart failure.

### Autophagy is up-regulated in high over-expressing line

**Figure 6:**
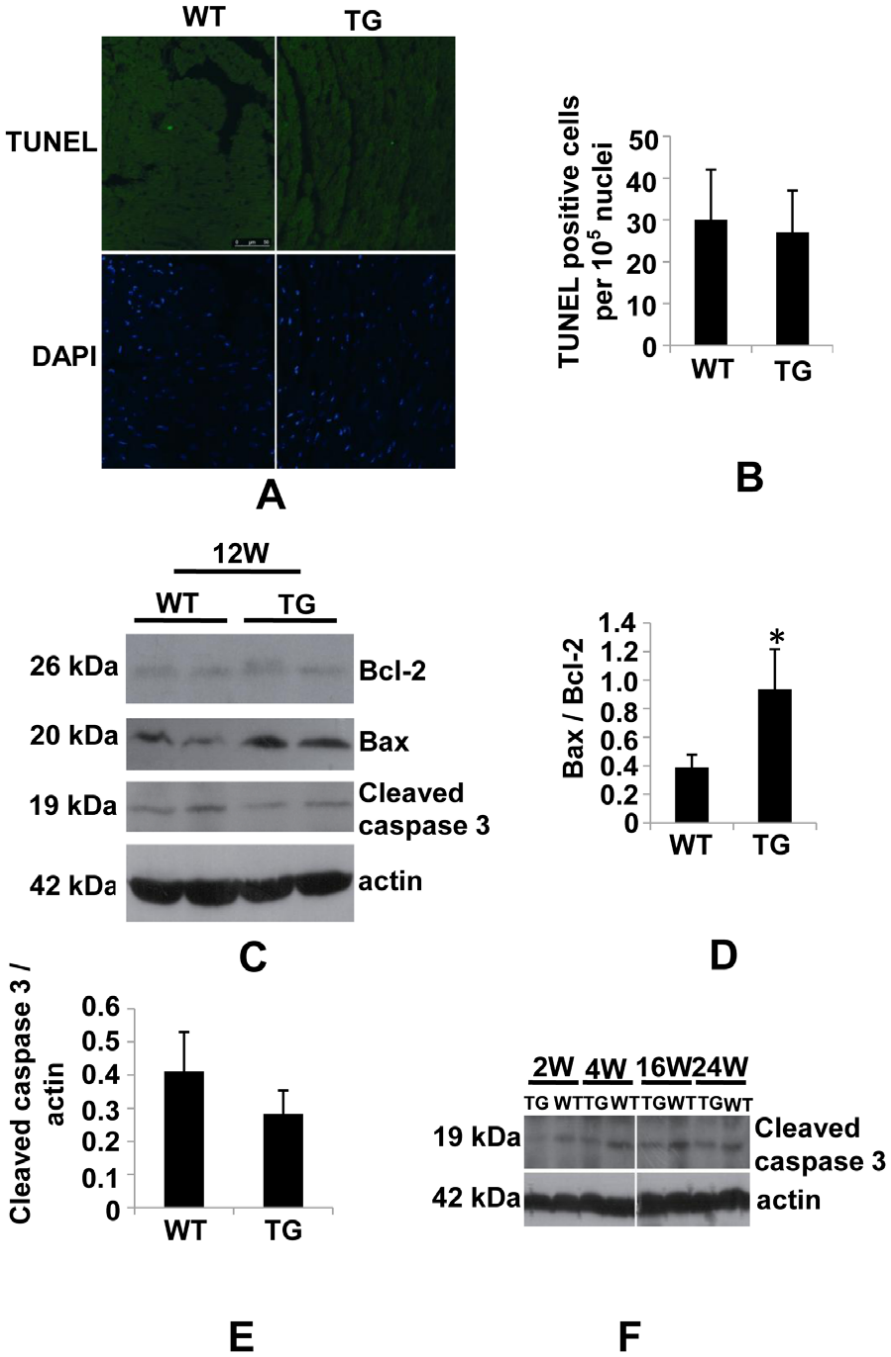
Assessment of apoptosis in high over-expressing line. (A), Representative images of TUNEL staining (green) and DAPI staining (blue) for nuclei on cryosectioned hearts from 3-month-old high over-expressing line and WT control mice. B, Quantification of TUNEL staining positive cells (30 microscopic fields from 3 mice for TG or WT). C, Representative western blot of apoptosis-related proteins Bcl-2, Bax and cleaved caspase 3 in heart extracts obtained from 3-month-old high over-expressing line and WT control mice. (D), Densitometric analysis of Bax and Bcl-2 immunoblots. (E), Densitometric analysis of cleaved caspase 3 immunoblots. (F), Representative western blot of time course analysis of cleaved caspase 3 in heart extracts obtained from high over-expressing line and WT control mice. *P<0.05, WT vs. TG.

**Figure 7:**
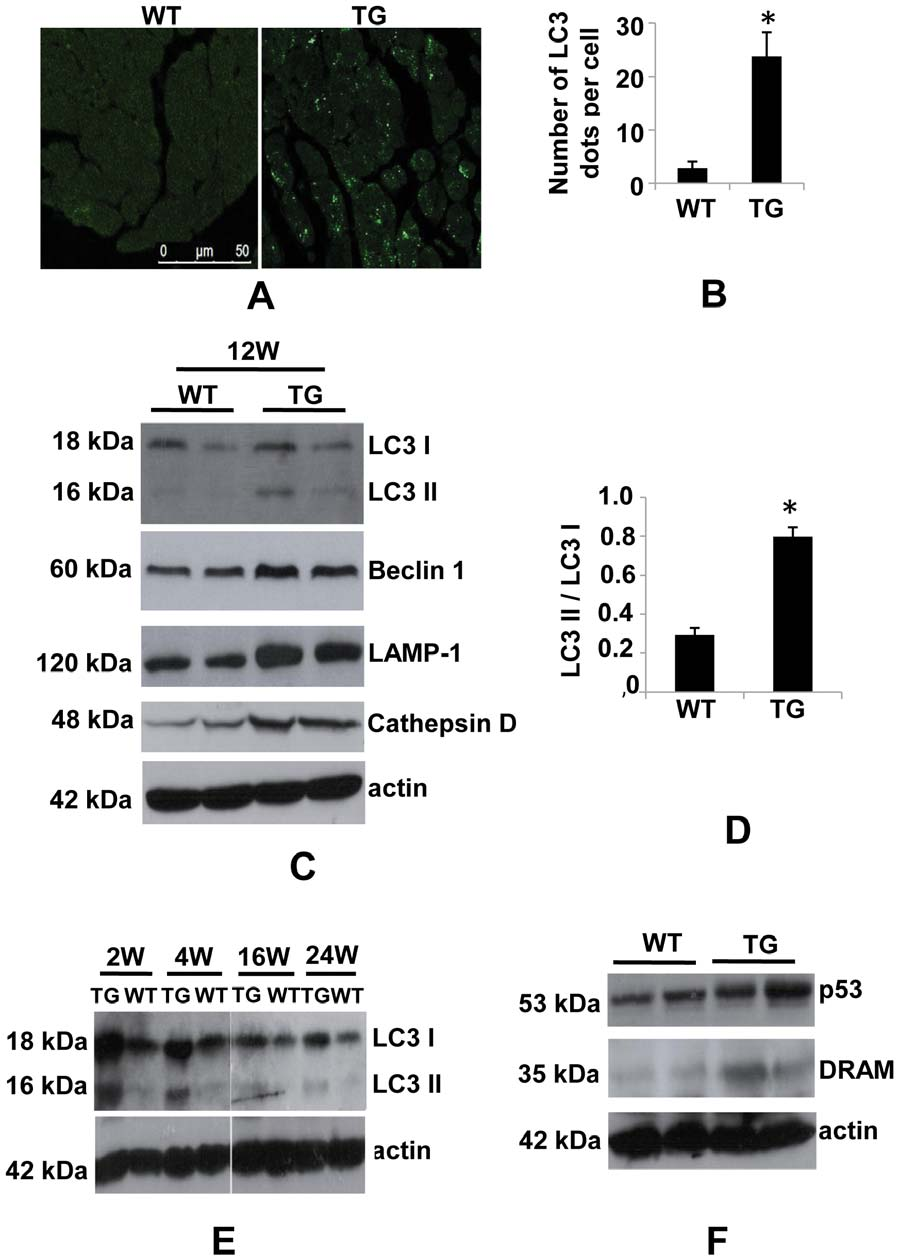
Assessment of autophagy in high over-expressing line. (A), Representative images of Immunostaining of LC3 on paraffin section of hearts from high over-expressing line and WT control mice. (B), Quantification of LC3 positive dots (For each group, 4 mice were studied). (C), Representative western blot of autophagy-related proteins LC3, Beclin 1, LAMP-1 and cathepsin D in heart extracts obtained from 3-month-old high over-expressing line and WT control mice. (D), Densitometric analysis of LC3 immunoblots. (E), Representative western blot of time course analysis of LC3 processing in heart extracts obtained from high over-expressing line and WT control mice. (F), Representative western blot of acetylated p53 and DRAM.

Previously, PDCD5 has been shown to suppress growth of multiple types of cancer cells by promoting apoptosis [2]–[4]. To examine whether PDCD5 induces apoptosis in heart, we performed TUNEL staining on cryosection of hearts from 3-month-old mice. Surprisingly, the number of TUNEL-positive cells remained unaltered in transgenic mice as compared to WT littermate control mice (Fig. 6A–B). We then conducted Western blot to detect apoptosis related gene expression. Bcl-2 didn't show significant change in transgenic mice, while Bax and Bax to Bcl-2 ratio were significantly up-regulated in transgenic hearts (Fig. 6C–D). However, Cleaved caspase 3, a downstream executor of apoptosis, was not significantly increased in transgenic mice of different ages as compared to littermate control (Fig. 6C, 6E–F). These data suggest that apoptosis is not likely the causative reason for dilated cardiomyopathy in high PDCD5 over-expressing line. As multiple lines of evidence suggest that autophagy is a major mechanism for certain forms of heart disease [18]–[20], we performed immunohistochemistry on paraffin section to detect autophagy associated protein LC3 II. As expected, LC3 puncta were dramatically increased in 3-month-old transgenic mice (Fig. 7A–B). Accordingly, western blot showed LC3 II to LC3 I processing, a molecular marker for autophagy, was significantly increased in transgenic mice (Fig. 7C–D). Lysosomal-associated membrane protein-1 (LAMP-1) and Cathepsin D, two markers for lysosome activity, were up-regulated in transgenic mice at 3 months of age (Fig. 7C), confirming the increased autophagic activity in transgenic mice. Furthermore, time course analysis by western blot using LC3 antibody showed that autophagic activity remained elevated in mice at 2 weeks to 6 months of age (Fig. 7E), suggesting the essential role of autophagy in the development of dilated cardiomyopathy.

To determine the potential mechanism of autophagy activation by PDCD5 over-expression, we examined the protein level of acetylated p53 and its downstream target damage-regulated autophagy modulator (DRAM). PDCD5 was shown to activate p53, which plays dual roles in autophagy depending on the context and subcellular localization [23]. Nucleus p53 can induce autophagy via transcriptional effects including transactivation of DRAM gene [24], while cytoplasmic p53 represses autophagy through poorly-understood mechanisms [25]. As shown in Fig. 7F, acetylated p53 at lysine 120 and DRAM were significantly up-regulated by PDCD5 over-expression in heart. Taken together, these data demonstrate that autophagy is significantly up-regulated possibly through p53 activation in high PDCD5 over-expressing transgenic mice. However, the precise molecular mechanisms of autophagy regulation by PDCD5 remain to be elucidated.

### Low PDCD5 over-expressing line is more susceptible to Ang II-induced cardiac hypertrophy

**Figure 8:**
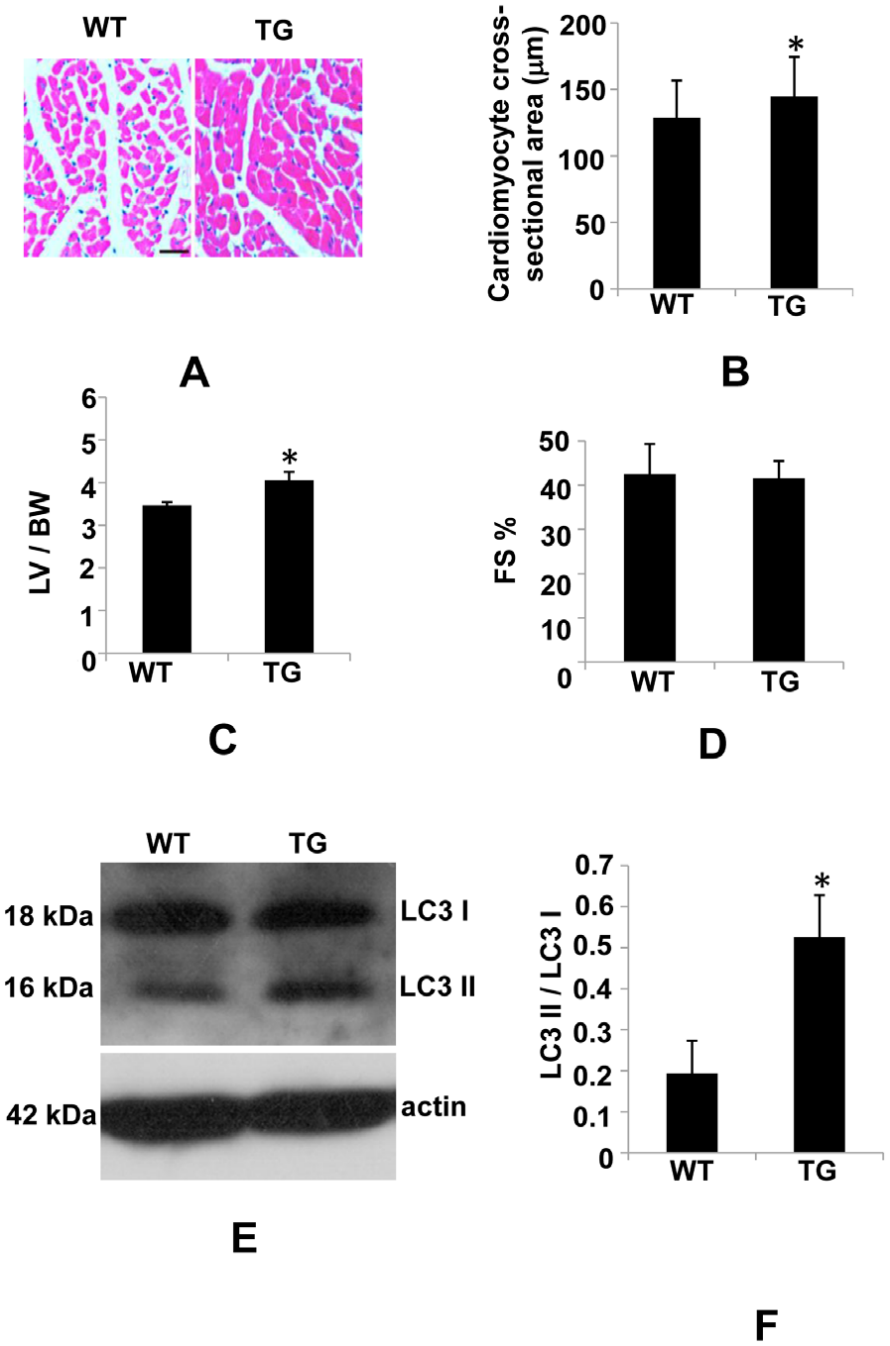
Susceptibility of low over-expressing line to Ang II-induced cardiac hypertrophy. (A), H&E staining on heart sections. Bar, 40 µm. (B), Measurement of two-dimensional cardiomyocyte cross-sectional area. (C), LV to BW ratio is significantly increased in Ang II- treated transgenic mice as compared to WT control mice receiving identical treatment. (D), In transgenic mice treated with Ang II, FS% is not significantly altered as compared to WT control mice. (E), Representative images of LC3 processing in Ang II-treated transgenic mice and WT control mice. (F) Densitometric analysis of LC3 immunoblots. *P<0.05, WT vs. TG (n = 4–5 for each group).

Given that PDCD5 is upregulated in hypertrophied heart and high over-expressing line develops dilated cardiomyopathy and heart failure, we hypothesized that low over-expressing line may be more susceptible to Ang II-induced cardiac hypertrophy. To test this hypothesis, we challenged the low over-expressing line with Ang II for 2 weeks. The Ang II-infused transgenic mice showed increased LV to BW ratio and enlarged cross-sectional area of individual cardiomyocyte as compared to WT control mice undergoing identical treatment (Fig. 8A–C). Systolic function measured as FS% was not significantly changed in Ang II-treated transgenic mice (Fig. 8D). These results suggest that low over-expressing line is predisposed to Ang II-induced cardiac hypertrophy without attenuating cardiac function. In addition, LC3 processing was increased in heart of Ang II-treated transgenic mice (Fig. 8E–F), implicating a role of autophagy in the susceptibility to Ang II-induced cardiac hypertrophy in transgenic mice.

## Discussion

The major findings in the present study are that (1) PDCD5 expression is significantly up-regulated in cardiac hypertrophy; (2) Cardiac specific high over-expression of hPDCD5 results in dilated cardiomyopathy, heart failure and premature sudden death; (3) Autophagy is dramatically up-regulated in transgenic mice with high hPDCD5 over-expression, which is associated with increased p53 activity; (4) Low PDCD5 over-expressing line is more susceptible to Ang II-induced cardiac hypertrophy.

### PDCD5 in dilated cardiomyopathy and heart failure

Previous study demonstrated that PDCD5 mRNA is expressed at an appreciable level in adult hearts [1], suggesting PDCD5 may play an important role in heart. We showed that PDCD5 was strongly induced upon Ang II treatment or transverse aortic constriction, which has never been studied before, indicating the potential role of PDCD5 in cardiac remodeling and cardiac function. Indeed, high PDCD5 over-expression (10 fold) led to autophagy activation, dilated cardiomyopathy and sudden death. In addition, autopsy revealed that heart from the transgenic founder died spontaneously was enlarged in both atria and ventricles. On the basis of the phenotypes we observed in the founder and high over-expression line, dilated cardiomyopathy and heart failure in transgenic mice were not likely caused by disruption of endogenous gene(s).

### Autophagy activation in cardiac remodeling

A growing body of evidence showed that autophagy is involved in the pathogenesis of cardiac diseases and plays distinct roles depending on the context and dosage [18]–[20]. For instances, cardiac autophagy is activated in response to pressure overload and contributes to the transition from stable cardiac hypertrophy to heart failure [29]. In ischemia-reperfusion injury, autophagy is up-regulated. In the phase of ischemia, autophagy is beneficial, while during perfusion autophagy is detrimental to the heart by triggering cardiomyocytes apoptosis [30]–[31]. In a desmin-related cardiomyopathy mouse model, autophagy is up-regulated and beneficial to the cardiac function [32]. A recent study demonstrated that autophagy contributes to the pathology of diabetic cardiomyopathy [33]. In our high PDCD5 over-expressing line, autophagy was dramatically up-regulated, with significant increase in autophagosomes, LC3 processing and cathepsin D, LAMP-1 protein abundance. Moreover, Beclin 1, a yeast atg6 homologue in mammalian cells [34], was up-regulated in PDCD5 transgenic heart. It has been indicated that increased autophagy accompanied by elevated Beclin 1 protein levels may cause cell death [35]–[36]. While we barely detected apoptotic cells, autophagy may be the major mechanism of the pathogenesis of dilated cardiomyopathy and heart failure in high over-expressing line. We cannot exclude the possibility that autophagy is adaptive at early stages of development of heart failure in our transgenic mice model. However, we find no evidence of accumulation of intracellular protein aggregates and poly-ubiquitinated proteins (Figure S5), which are normally toxic and protected by induced autophagy in heart [32]–[33], [37]–[39]. In low over-expressing line, autophagy remained unchanged at all ages examined except mice at 2 months of age (Figure S6A). Beclin 1 wasn't elevated in the low-expressing line (Figure S6B). However, autophagy is up-regulated in response to Ang II treatment in low over-expressing line, indicating its involvement in cardiac remodeling. Considering Ang II-treated low over-expressing line manifests no abnormalities of cardiac function, the increased autophagy may be adaptive. Indeed, Ang II-treated low over-expressing line shows less autophagy than high over-expressing line, consistent with the notion that autophagy beyond certain threshold is detrimental [19], [21]. However, increased autophagic activity could be maladaptive and cardiac function could be deteriorated in low over-expressing line in response to long-term Ang II infusion. This remains to be determined by future study.

### p53 and autophagy

One remaining question is how autophagy is activated in high PDCD5 over-expressing line and Ang II-treated low over-expressing line, but not normal low over-expressing line. PDCD5 is initially identified as a tumor suppressor, which inhibits cell growth in a variety of cancer cells by inducing apoptosis [2]–[3]. In cancer cells, PDCD5 interacts with Tip60, which activates p53, a regulator playing dual roles in autophagy depending on the context and subcellular localization [20]–[21]. Nucleus p53 function as a transcription factor inducing autophagy by trans-activating autophagy related genes. On the other hand, cytoplasmic p53 represses autophagy through poorly-characterized mechanisms [30]–[32]. Thus, p53 may be involved in autophagy activation in our transgenic mice model. Indeed, acetylated p53 at lysine 120 was increased in high over-expressing line. Moreover, DRAM, a p53 downstream target, was up-regulated in high over-expressing line, Thus, PDCD5 may induce autophagy via activating p53 in a dose-dependent way. Additional work is required to establish the direct link between PDCD5 level and degree of p53-mediated autophagy activation. Interestingly, despite elevated p53 protein abundance and increase in Bax/Bcl-2 ratio, an index of cell susceptibility to apoptosis [40], we barely detected apoptotic nuclei with TUNEL staining assay in high over-expressing line. Consistent with this finding, cleaved caspase 3, a major downstream apoptosis executor, was not significantly increased. The lack of apoptosis may be attributed to the incomplete apoptotic program in cardiomyocyte [41]–[42].

### Conclusion

In conclusion, PDCD5 is upregulated in the development of cardiac hypertrophy. We generated cardiac specific PDCD5 transgenic mice to determine the role of PDCD5 in cardiac remodeling and function. High over-expression of human PDCD5 leads to dilated cardiomyopathy and sudden death. This is attributed at least in part to elevated autophagic activity, which is associated with increased activity of p53. Low over-expression of human PDCD5 is more susceptible to Ang II-induced cardiac hypertrophy. It will be interesting to further examine the function and decipher the precise molecular mechanisms of PDCD5 in cardiac hypertrophy and heart failure using loss-of-function method. It also will be interesting to investigate the function of PDCD5 in other cardiac diseases such as ischemic heart disease.

## Materials and Methods

### Ethics Statement

All animal experimentation in this study was conducted in accordance with the National Research Council Guide for Care and Use of Laboratory Animals, with the approved protocols by the Institutional Animal Care and Use Committee of Shanghai, China [SYXK (SH) 2011-0112]. Efforts were made to minimize suffering.

### Reagents

Mouse anti actin (Sigma), rabbit anti LC3 (MBL), rabbit anti Bcl-2 (Cell Signaling), mouse anti acetylated p53 (Abcam), rat anti LAMP-1 (Santa Cruz), rabbit anti Cathepsin D (Santa Cruz), rabbit anti cleaved caspase 3 (Cell Signaling), rabbit anti Bax (Cell Signaling), rabbit anti DRAM (Abcam), Cy3-conjugated goat anti rabbit (Jackson ImmunoResearch), cy3-conjugated goat anti-mouse (Jackson ImmunoResearch), rat anti-HA (Roche Applied Sciences), rabbit anti Beclin 1(MBL), rabbit anti ubiquitin (Sigma).

### Chronic Ang II infusion

Eight-week-old C57BL/6 mice were infused with Ang II (n = 15) or saline (n = 12) for 14 days via osmotic minipumps (model 2002; Alzet). The osmotic minipumps were implanted subcutaneously, slightly posterior to the scapula. Ang II was dissolved in 0.5 mol/l NaCl and 1 mmol/l acetic acid and infused at a rate of 2.16 mg/kg/day.

### Transverse aortic constriction (TAC)

Seven-week-old male mice were subjected to transverse aortic constriction (TAC) for 2 weeks as described previously [29], [43].

### Transgenic mice and genotyping

Human influenza hemagglutinin (HA) epitope tagged full length human PDCD5 cDNA was PCR amplified from the expressing construct PCDB-PDCD5 and cloned into alpha myosin heavy chain promoter plasmid. The plasmid was digested with Not I, generating a fragment containing the α-MHC promoter, HA-tagged hPDCD5 cDNA, and human growth hormone polyA, which was purified and microinjected into the pronuclei of C57BL/6 mouse eggs. The eggs were then implanted into the oviducts of pseudo-pregnant recipients. Genotyping for Transgenic founders were performed by PCR analysis of mouse tail DNA and the primers used were as follows: forward primer; 5′-GGCGCTGAGGAGACAGAG-3′, reverse primer: 5′-GTCTTCCGTACAGGATGGACGTG-3′. Five founders identified by PCR genotyping were bred with C57BL/6 mice. Generation of transgenic mice was performed in Model Animal Research Center of Nanjing University.

### Western blot

Mouse hearts were homogenized in lysis buffer and the lysates were collected and quantified with Nanodrop®. Proteins were separated in a SDS-PAGE gel and transferred to nitrocellulose membrane. The membrane was then blocked for 1 hour at room temperature with 5% non fat milk in tris buffered saline containing 0.1% Tween (TBST), probed with primary antibody overnight at 4°C, washed with TBST three times, and incubated with secondary antibody for 1 hour at room temperature, washed again with TBST three times. Immunolabeled proteins were then revealed by using ECL Plus (GE Healthcare).

### Histology

Mouse hearts were perfusion washed with PBS and fixed with ice cold 4% paraformaldehyde in PBS. Hearts embedded in paraffin were cut into serial 5 µm sections (Leica RM2255 rotary microtome) and stained with H&E or Masson trichrome to evaluate morphology and cellular dimensions. Hearts for TUNEL staining were cryoprotected in 30% sucrose overnight and embedded in freezing matrix. Serial 8 µm cryostat sections were prepared (Leica CM3050S cryostat) and stored at −80°C until use. TUNEL assays were performed with the In Situ Cell Death Detection Kit (Roche Applied Science) according to the manufacturer's instructions. The sections were counterstained with DAPI.

### Immunostaining

Tissues were permeabilized with 0.3% Triton X-100 in 1× PBS (PBST) for 30 min and then blocked with 0.3% horse serum in BSA in PBST for 1 h. Sample were incubated overnight with primary antibody at 4°C, washed with PBST 3 times, and incubated with cy3-conjugated goat anti-rabbit IgG for 1 hour at room temperature in the dark and were washed with PBST. DAPI staining was performed as counterstaining.

### Cardiomyocyte cross-sectional area

The cross-sectional area of cardiomyocytes was measured by H&E staining with image analysis software (Image J) and calculated as the mean of 120–150 cells from randomly selected fields.

### RNA isolation and Real-Time RT-PCR

Total RNA was prepared from hearts of WT and transgenic mice using TRIZOL® reagent, and cDNA was synthesized using Superscript III® reverse transcriptase (RT) (Invitrogen). mRNA expression was analyzed by quantitative Real-Time RT-PCR (Applied Biosystems StepOne Plus™) and were normalized to GAPDH housekeeping gene. Real-time RT-PCR was performed in triplicate for each sample. The primers used for Real-Time RT-PCR are shown in Table 1.

**Table 1:** Oligos for Real-Time RT-PCR experiments.

Gene	Primer	Sequence
Mouse ANF	Forward	5′-GTACAGTGCGGTGTCCAACA-3′
	Reverse	5′-TCTCCTCCAGGTGGTCTAGCA-3′
Mouse BNP	Forward	5′-CACCGCTGGGAGGTCACT-3′
	Reverse	5′-GTGAGGCCTTGGTCCTTCAA-3′
Mouse SAA	Forward	5′-AGCAGATGTGGATCACCAAG-3′
	Reverse	5′-CTGCAACCACAGCACGATTG-3′
Mouse β-MHC	Forward	5′-GCATTCTCCTGCTGTTTCCTT-3′
	Reverse	5′-TGGATTCTCAAACGTGTCTAGTGA-3′
Mouse GAPDH	Forward	5′-AAGAAGGTGGTGAAGCAG-3′
	Reverse	5′-TCATACCAGGAAATGAGC-3′
Mouse SERCA	Forward	5′-GGTGTGCAGCCAGCTGTTCC-3′
	Reverse	5′-GCTGTGAGAAGCTGTGAGCA-3′
Human PDCD5	Forward	5′- GTGATGCGGCCCAACAG-3′
	Reverse	5′-ATCCAGAACTTGGGCTAAGATACTG-3′
Mouse α-MHC	Forward	5′-GGAAGAGTGAGCGGCGCATCAAGG-3′
	Reverse	5′-CTGCTGGAGAGGTTATTCCTCG-3′

### Echocardiography

Echocardiography was performed on mice anesthetized with isoflurane. Echocardiographic imaging was conducted using a Vevo 770 platform (VisualSonics, Canada). Measurements were carried out at least in triplicate. The parameters measured were as follows: Heart rate, LVID;d; LVID;s; left ventricular posterior wall thickness (diastole) (LVPW;d) and EF. FS% was calculated as follows: FS% = (LVID;d–LVID;s)/LVID;d×100%.

### Statistics

All values were expressed as mean ± SD. Data were analyzed using Student's *t*-test and significance was considered with *P*<0.05.

## Supporting Information

Figure S1 **Up-regulation of PDCD5 in acute pressure overload-induced cardiac hypertrophy.** (A), HW∶BW ratio showing significant increase in cardiac mass in mice subjected to TAC for 2 weeks compared to sham-operated control mice. (B), Representative western blot of PDCD5 and internal control actin proteins in heart extracts from 7–week-old male mice subjected to TAC or sham surgery.
(TIF)

Figure S2 **Enhanced PDCD5 mRNA level in heart from high over-expressing line.** PDCD5 mRNA was determined by quantitative Real-Time RT-PCR analysis in cDNA samples derived from heart of transgenic line 32 and WT control mice. Expression levels were normalized to GAPDH. Experiments were performed twice in triplicate with similar results. *P<0.05, WT vs. TG.
(TIF)

Figure S3 **Characterization of low over-expressing line.** (A) Representative western blot of hPDCD5 protein in heart extracts from high and low over-expressing line. (B), Hearts from low over-expressing line exhibiting similar size as compared to non transgenic littermate control mice (6 months old). (C), HW∶BW ratio of 6-month-old mice showing no significant difference in cardiac mass between low PDCD5 expressing line as compared to the non transgenic littermate control mice (n = 7 for TG, n = 6 for WT).
(TIF)

Figure S4 **Enlarged heart from 3-week-old high over-expressing line.** (A), High over-expression of hPDCD5 in the transgenic mice results in enlarged hearts. (B), Hematoxylin and eosin staining of the heart from high over-expressing line and non-transgenic littermate control mice.
(TIF)

Figure S5 **Detection of accumulation of protein aggregates in high over-expressing line.** (A), Representative immunohistochemistry images of time course analysis of ubiquitinated protein aggregates in heart section from high over-expressing line and WT control mice. (B), Representative western blot of time course analysis of poly-ubiquitinated protein in heart extracts obtained from high over-expressing line and WT control mice.
(TIF)

Figure S6 **Autophagy in low over-expressing line.** (A), Representative western blot of time course analysis of LC3 processing in heart extracts obtained from low over-expressing line and WT control mice. (B), Representative western blot of time course analysis of Beclin 1 protein in heart extracts obtained from low over-expressing line and WT control mice.
(TIF)

Table S1 Echocardiographic data for low over-expressing line. Results from echocardiography in low over-expressing line and WT control. Data presented as mean± SD, n = 5 in each group. LVID;d, left-ventricular internal diameter at diastole; LVPW;d, left-ventricular posterior wall at diastole; LVID;s, left-ventricular internal diameter at systole; LVPW;s, left-ventricular posterior wall at systole; LVAW;d, left ventricular anterolateral wall at diastole; LVAW;s, left ventricular anterolateral wall at systole; FS%, percent fractional shortening; EF, ejection fraction.
(DOC)
