# Supplementary figures and images for: Involvement of Autophagy in Cardiac Remodeling in Transgenic Mice with Cardiac Specific Over-Expression of Human Programmed Cell Death 5
Source: PLoS One. 2012 Jan 11;7(1):e30097. doi: 10.1371/journal.pone.0030097 (PMC3256219; doi:10.1371/journal.pone.0030097)

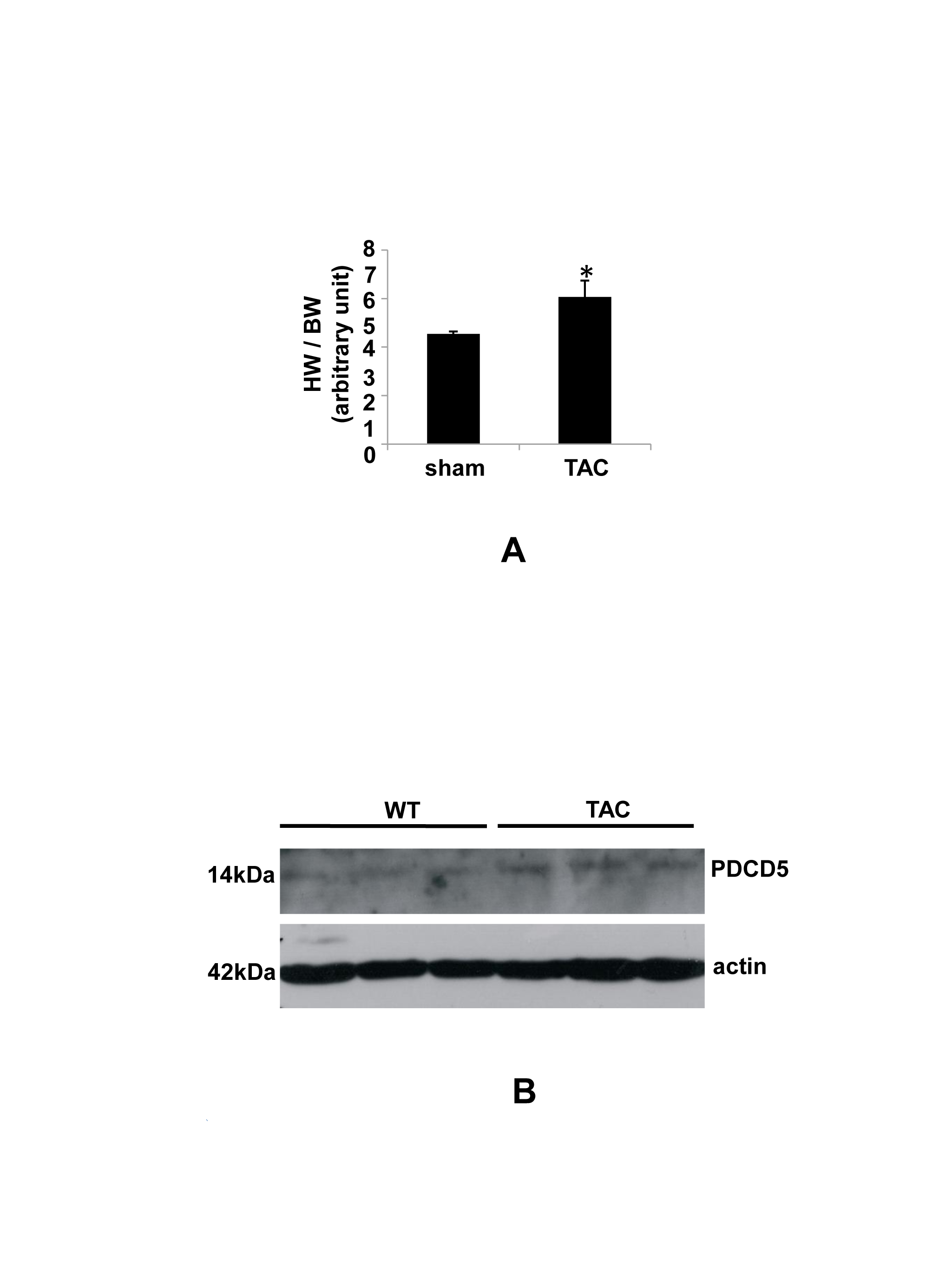

Supplement: Figure S1 — Up-regulation of PDCD5 in acute pressure overload-induced cardiac hypertrophy. (A), HW∶BW ratio showing significant increase in cardiac mass in mice subjected to TAC for 2 weeks compared to sham-operated control mice. (B), Representative western blot of PDCD5 and internal control actin proteins in heart extracts from 7–week-old male mice subjected to TAC or sham surgery. (TIF) [file pone.0030097.s001.tif]

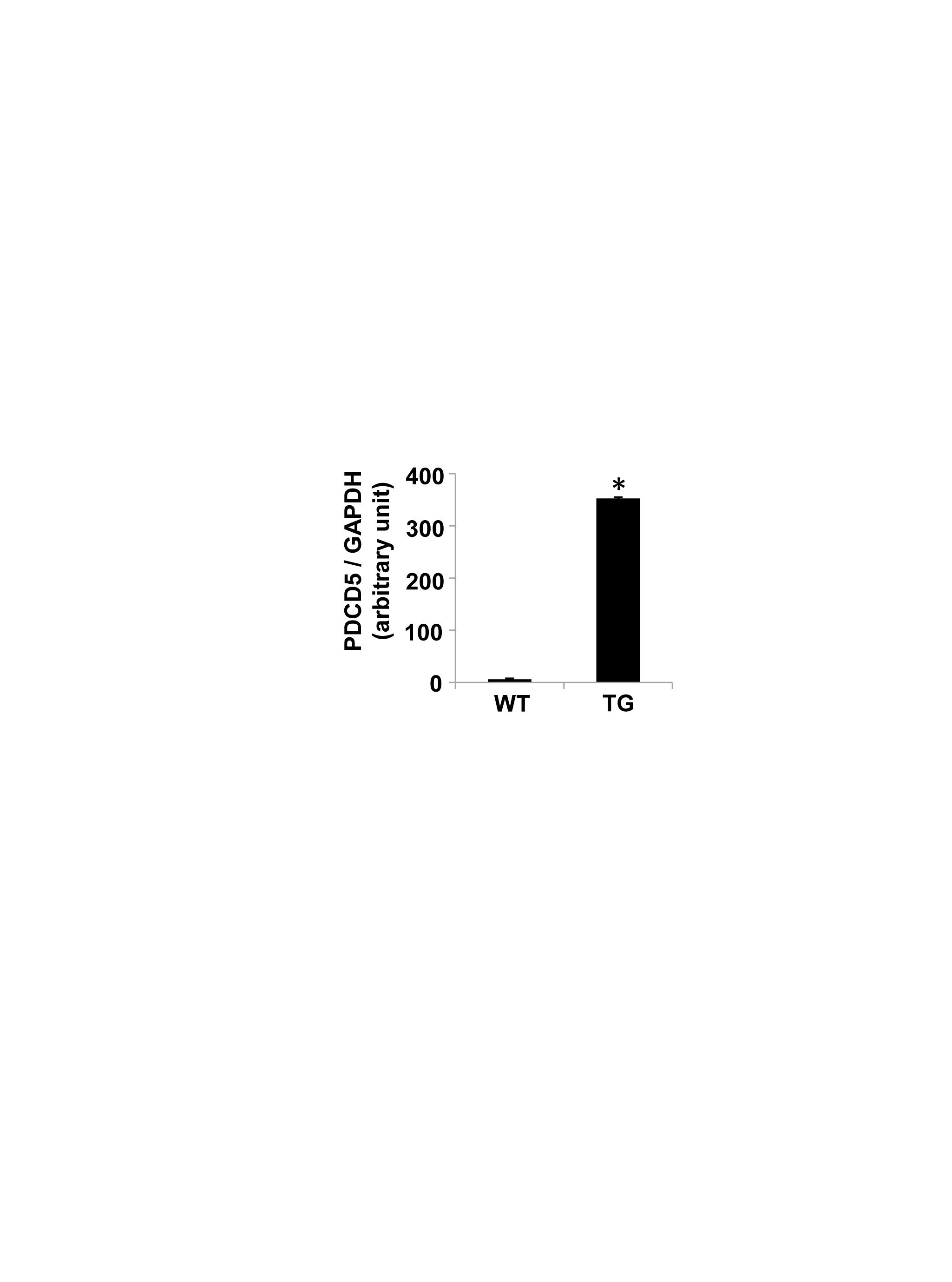

Supplement: Figure S2 — Enhanced PDCD5 mRNA level in heart from high over-expressing line. PDCD5 mRNA was determined by quantitative Real-Time RT-PCR analysis in cDNA samples derived from heart of transgenic line 32 and WT control mice. Expression levels were normalized to GAPDH. Experiments were performed twice in triplicate with similar results. *P<0.05, WT vs. TG. (TIF) [file pone.0030097.s002.tif]

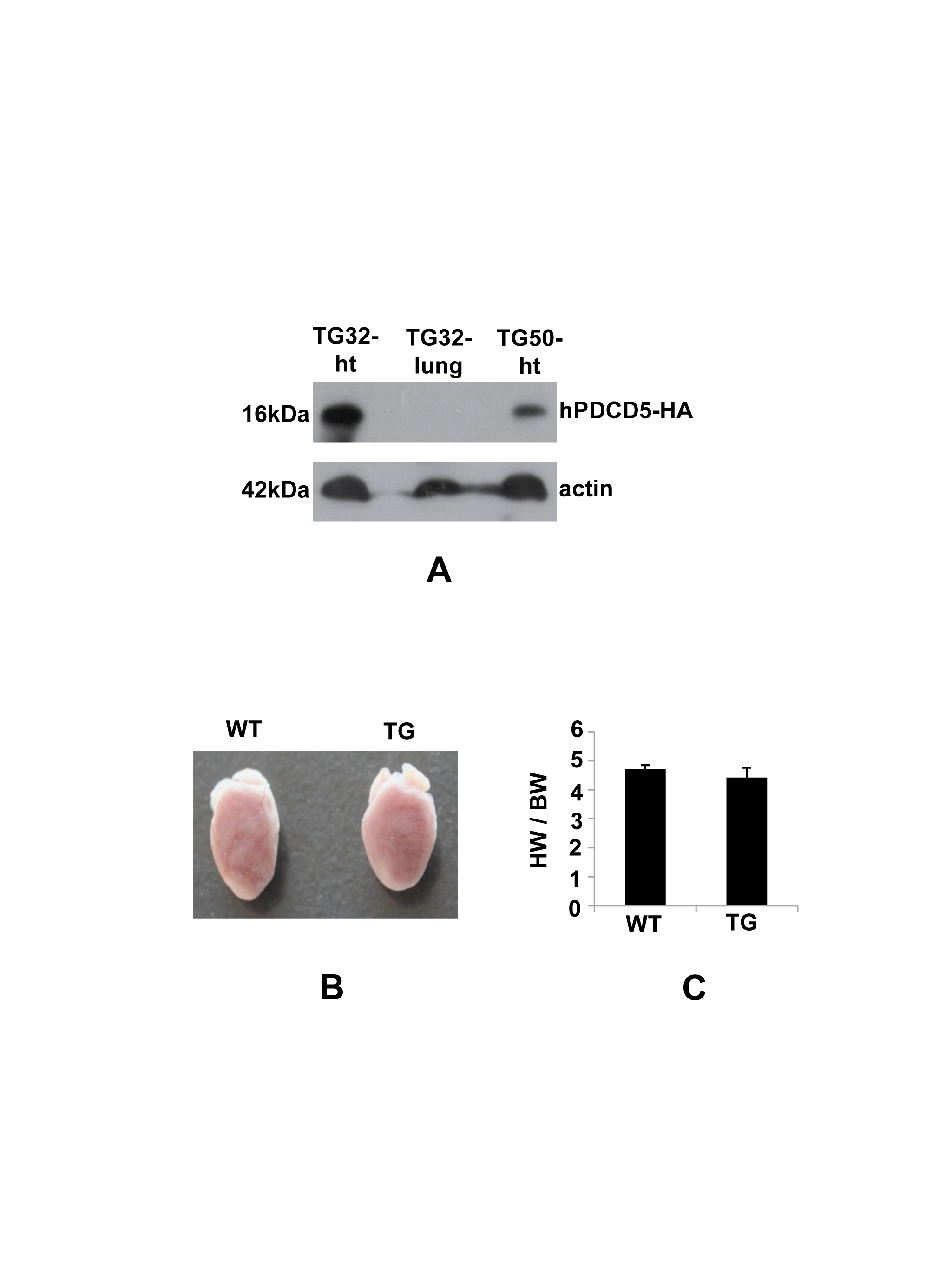

Supplement: Figure S3 — Characterization of low over-expressing line. (A) Representative western blot of hPDCD5 protein in heart extracts from high and low over-expressing line. (B), Hearts from low over-expressing line exhibiting similar size as compared to non transgenic littermate control mice (6 months old). (C), HW∶BW ratio of 6-month-old mice showing no significant difference in cardiac mass between low PDCD5 expressing line as compared to the non transgenic littermate control mice (n = 7 for TG, n = 6 for WT). (TIF) [file pone.0030097.s003.tif]

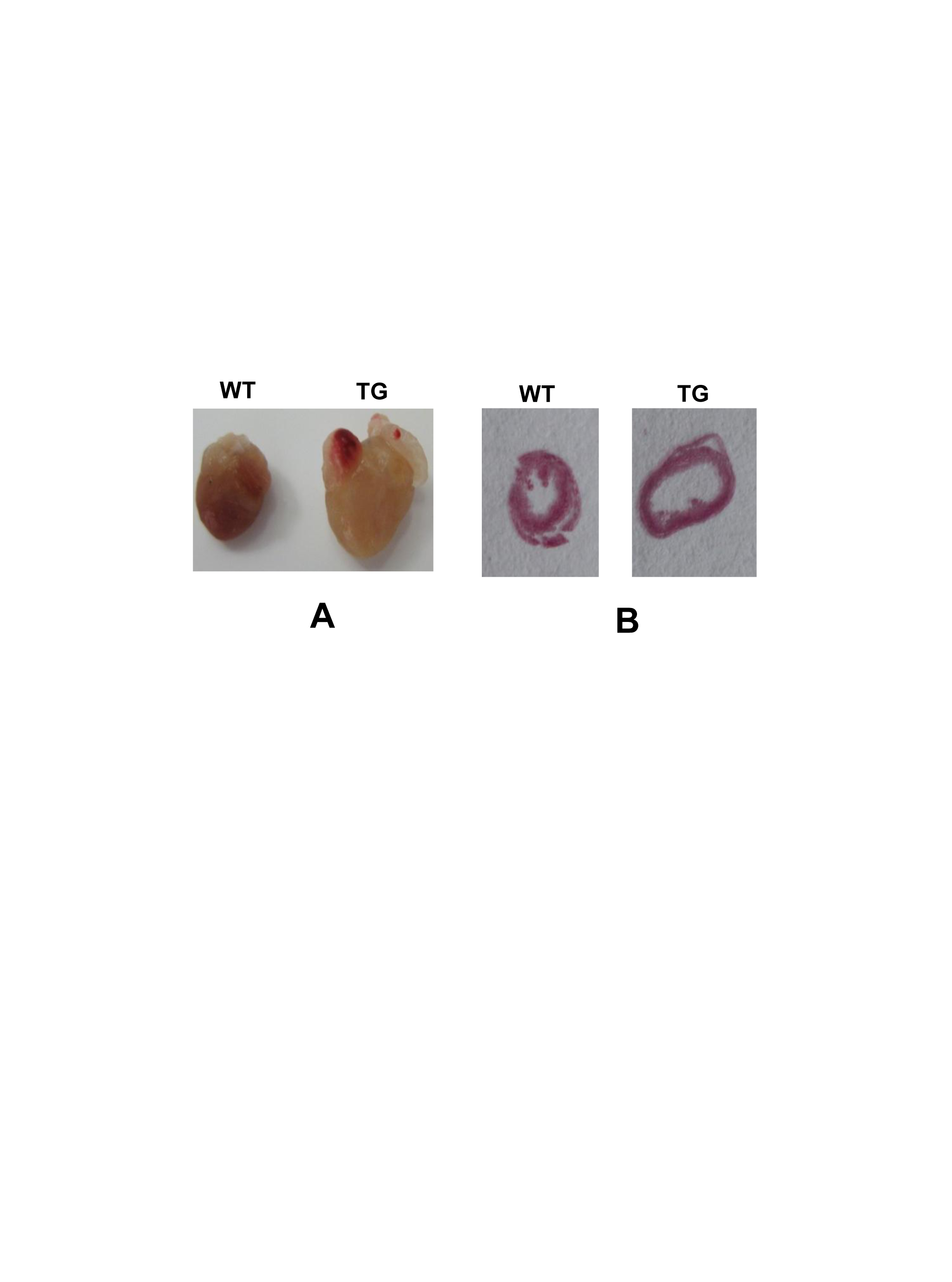

Supplement: Figure S4 — Enlarged heart from 3-week-old high over-expressing line. (A), High over-expression of hPDCD5 in the transgenic mice results in enlarged hearts. (B), Hematoxylin and eosin staining of the heart from high over-expressing line and non-transgenic littermate control mice. (TIF) [file pone.0030097.s004.tif]

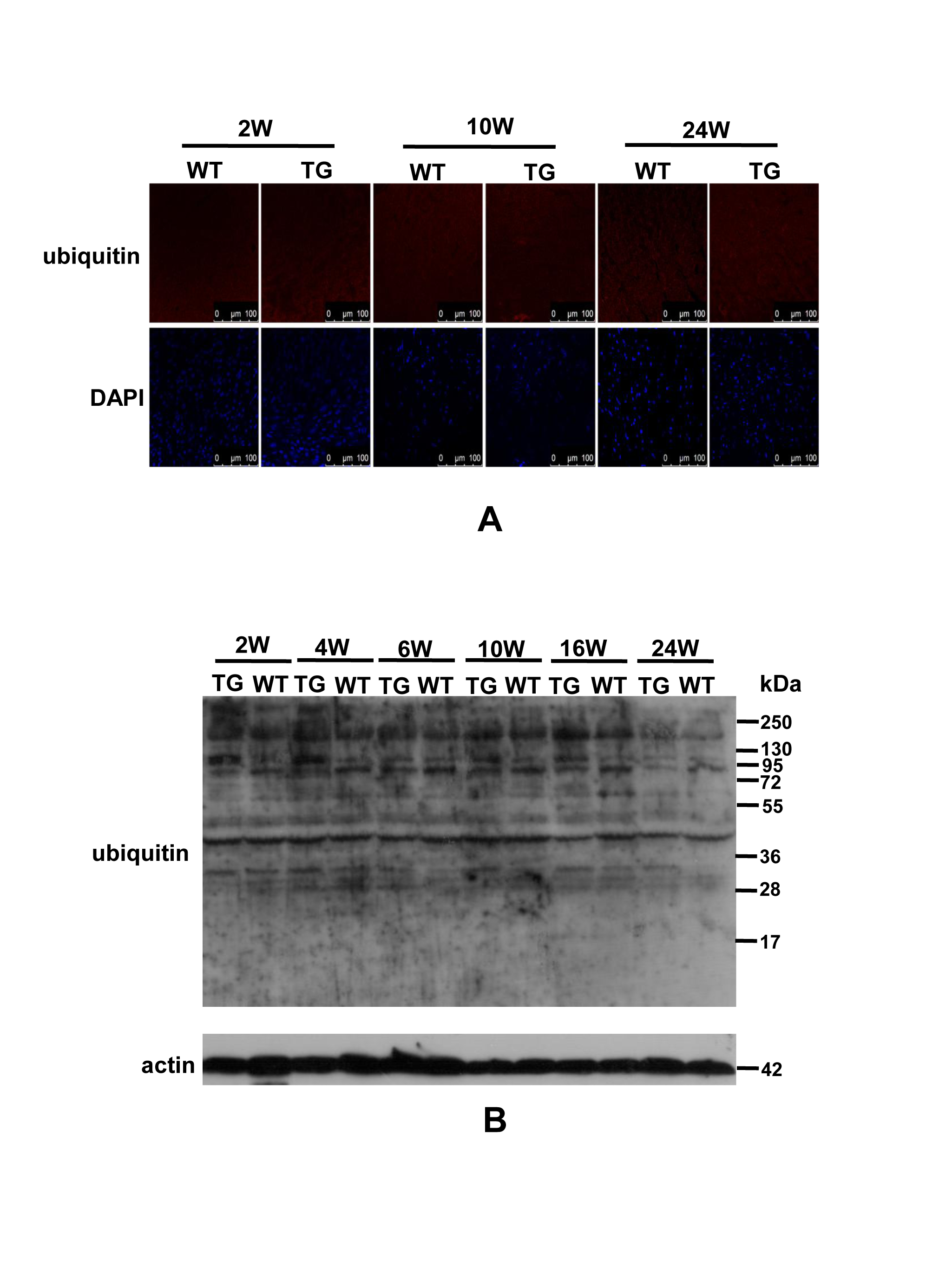

Supplement: Figure S5 — Detection of accumulation of protein aggregates in high over-expressing line. (A), Representative immunohistochemistry images of time course analysis of ubiquitinated protein aggregates in heart section from high over-expressing line and WT control mice. (B), Representative western blot of time course analysis of poly-ubiquitinated protein in heart extracts obtained from high over-expressing line and WT control mice. (TIF) [file pone.0030097.s005.tif]

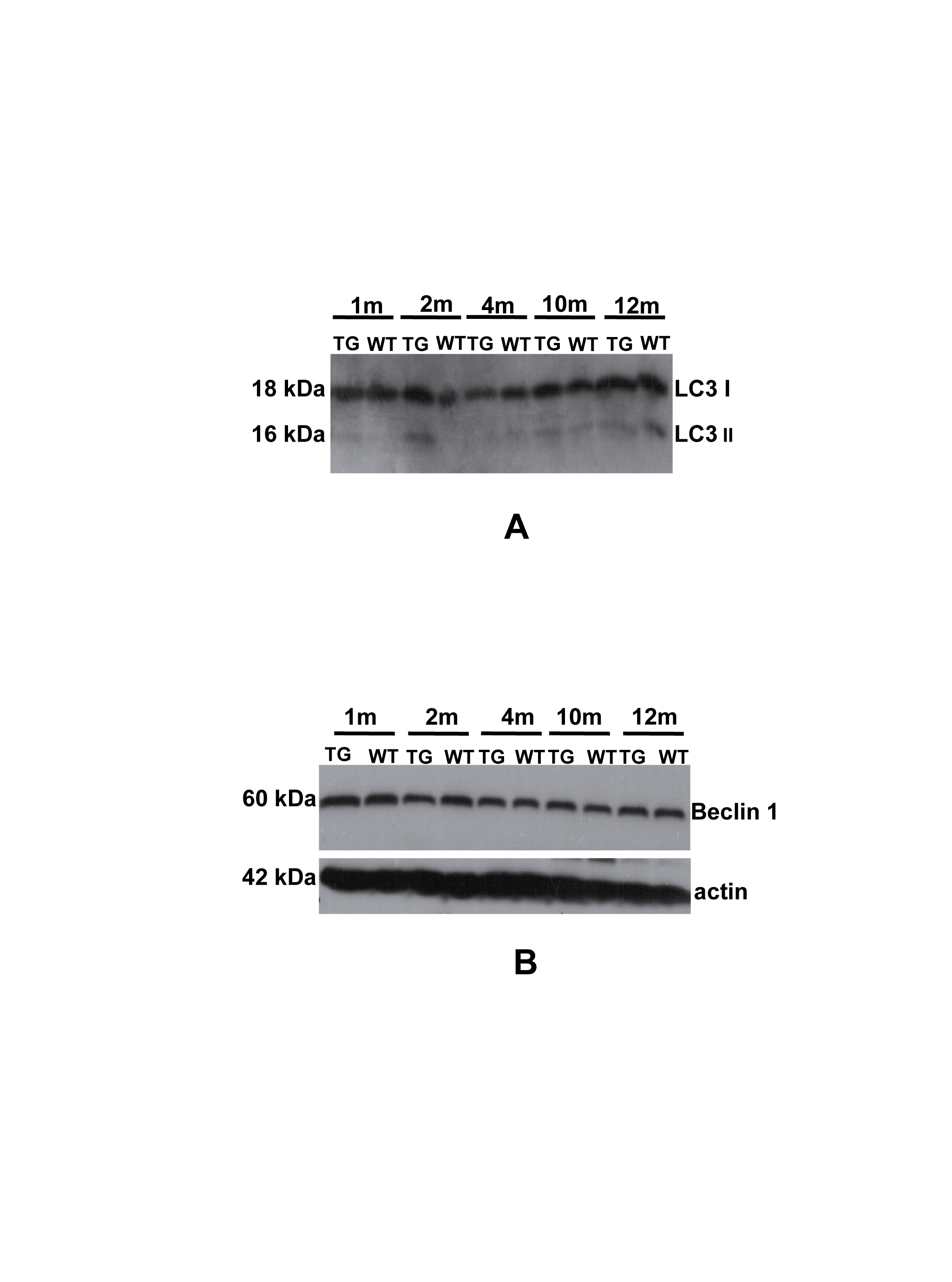

Supplement: Figure S6 — Autophagy in low over-expressing line. (A), Representative western blot of time course analysis of LC3 processing in heart extracts obtained from low over-expressing line and WT control mice. (B), Representative western blot of time course analysis of Beclin 1 protein in heart extracts obtained from low over-expressing line and WT control mice. (TIF) [file pone.0030097.s006.tif]
