# Supplementary material for: Involvement of Autophagy in Cardiac Remodeling in Transgenic Mice with Cardiac Specific Over-Expression of Human Programmed Cell Death 5
Source: PLoS One. 2012 Jan 11;7(1):e30097. doi: 10.1371/journal.pone.0030097 (PMC3256219; doi:10.1371/journal.pone.0030097)
Supplement: Table S1 — Echocardiographic data for low over-expressing line. Results from echocardiography in low over-expressing line and WT control. Data presented as mean± SD, n = 5 in each group. LVID;d, left-ventricular internal diameter at diastole; LVPW;d, left-ventricular posterior wall at diastole; LVID;s, left-ventricular internal diameter at systole; LVPW;s, left-ventricular posterior wall at systole; LVAW;d, left ventricular anterolateral wall at diastole; LVAW;s, left ventricular anterolateral wall at systole; FS%, percent fractional shortening; EF, ejection fraction. (DOC) [file pone.0030097.s007.doc]

**Table S1**. Echocardiographic data for low over-expressing line

| **Parameter** | **WT** | **TG** |
| --- | --- | --- |
| LVID; d | 3.54±0.32 | 3.66±0.06 |
| LVPW; d | 0.69±0.13 | 0.6±0.1 |
| LVID; s | 2.42±0.31 | 2.53±0.21 |
| LVPW; s | 1.03±0.13 | 1.01±0.09 |
| LVAW; d | 0.66±0.05 | 0.61±0.10 |
| LVAW; s | 0.98±0.09 | 0.94±0.17 |
| FS% | 31.6±3.3 | 31.1±4.7 |
| EF | 60.5±5 | 59.6±6.8 |
